# Human Amnion-Derived MSCs Alleviate Acute Lung Injury and Hinder Pulmonary Fibrosis Caused by Paraquat in Rats

**DOI:** 10.1155/2022/3932070

**Published:** 2022-03-19

**Authors:** Liming Gong, Xiuxuan Wang, Shaohua Xu, Futuan Liao, Manhong Zhou

**Affiliations:** ^1^Division of Endocrinology and Metabolism, Sichuan University, Chengdu, 610000 Sichuan, China; ^2^Reproductive Medicine Center, Affiliated Hospital of Zunyi Medical University, Zunyi Medical University, Zunyi, 563000 Guizhou, China; ^3^Emergency Department, Nankai University School of Medicine, Tianjin 300071, China; ^4^Emergency Department, Affiliated Hospital of Zunyi Medical University, Zunyi Medical University, Zunyi, 563000 Guizhou, China

## Abstract

**Methods:**

First, the purity of hAD-MSCs was determined by morphological observation and FCM, and the effects on the survival of paraquat-poisoned Sprague-Dawley rats were observed. All rats were randomly divided into three groups, defined as the sham control group (*n* = 8), model group (*n* = 15), and hAD-MSC-transplanted group (*n* = 17). Pneumonocyte damage and inflammatory cell infiltration were investigated in the three groups of rats, untreated control, paraquat only, and paraquat+hAD-MSC transplanted, using H&E staining. Fibrosis was investigated in three groups of rats using Masson's trichrome staining and Sirius red staining. The profibrotic factor TGF-*β*1, the composition of fibrotic collagen HYP, and the hAD-MSC-secreted immunosuppressive factor HLA-G5 in serum were investigated in the three groups of rats using ELISA. Furthermore, the distribution of hAD-MSCs was investigated in the three groups of rats using immunohistochemistry and hematoxylin staining.

**Results:**

The hAD-MSCs exhibited typical hallmarks of MSCs, improved the state of being and survival of paraquat-poisoned rats, reduced both lung injury and inflammation, and inhibited the progression of pulmonary fibrosis by decreasing the deposition of collagen and the secretion of both TGF-*β*1 and HYP. The hAD-MSCs could survive in damaged lungs and secreted appropriate amounts of HLA-G5 into the serum.

**Conclusion:**

The obtained results indicate that hAD-MSCs used to treat paraquat-induced lung injury may work through anti-inflammatory and immunosuppressive pathways and the downregulation of profibrotic elements. This study suggests that the transplantation of hAD-MSCs is a promising therapeutic approach for the treatment of paraquat-intoxicated patients.

## 1. Introduction

Paraquat (PQ) is a fast-acting herbicide used as a defoliating agent worldwide. PQ poisoning can damage multiple organs and tissues, and the most significant damage occurs to lung tissue. This is mainly due to the uptake and accumulation of paraquat by lung tissue [1]. According to some studies, we found that the mechanism of paraquat-induced lung injury mainly includes oxidative [2], alveolar [3, 4], mitochondrial [5], lipid [6], and chromosomal [7] damage, all of which can lead to acute lung injury (ALI). It results in alveolar epithelial cell damage and alveolar hemorrhage and edema, later showing irreversible pulmonary fibrosis [8]. However, there are individual differences in the time of pulmonary fibrosis formation, and it is positively correlated with dose of ingestion. Usually, pulmonary fibrosis will form approximately 3 days after moderate or severe acute paraquat poisoning, and the degree of pulmonary fibrosis will reach its peak about 14-28 days [9, 10]. PQ is extremely deadly even at small doses. Selective accumulation of PQ occurs in the lung, and there is no effective clinical therapy [11]. Although China has banned the production and use of PQ, it is still frequently encountered in cases of suicide and in outpatients who ingested PQ involuntarily, either through occupational or nonoccupational exposure. The exact mechanism of toxicity is not yet understood, and current therapies for PQ-induced lung fibrosis include cytoprotection, anti-inflammation, immunosuppression, and antioxidant administration [12–14].

Mesenchymal stem cells (MSCs) are capable of self-renewal and have immunosuppressive, anti-inflammatory, and antifibrotic properties [15]. Recent studies suggest that MSCs derived from bone marrow, umbilical cord, and adipose tissue have therapeutic applications in a wide range of lung diseases including bleomycin-induced pulmonary fibrosis, lipopolysaccharide- (LPS-) induced acute lung injury, and hypochlorous acid- (HOCL-) induced lung injury, among others [16–19]. Little attention has been focused on the treatment of PQ-induced lung injury or fibrosis with MSCs. Clinical application of MSCs derived from the tissues indicated above is limited by the timeliness of acquisition, the number of cells that can be harvested, and ethics [5, 19–21].

When compared to MSCs derived from other tissues, human amnion-derived mesenchymal stem cells (hAD-MSCs) have the following advantages: low immunogenicity, noninvasivity, simple acquisition, and relatively few ethical issues [22]. Recently, amnion mesenchymal stem cells (AM-MSCs), bone marrow MSCs, and human amniotic epithelial cells were tested as treatments in C57BL/6 mice exhibiting a repeat-dose bleomycin-induced model of lung injury [23]. This study demonstrated a significant decrease in inflammatory cell infiltration and a reduction in IL-1*β*, IL-6, TNF-*α*, collagen deposition, and profibrogenic cytokine TGF-*β* in the lung following treatment with AM-MSCs. The results suggest that AM-MSCs might be effective in lung injury. However, the study did not provide information on survival, and animal models of lung injury were not prepared for paraquat poisoning. Furthermore, the dose and timing of MSC injection were not suitable for preclinical studies or clinical application of lung injury caused by paraquat poisoning. Therefore, the main purpose of this experiment is to investigate whether hAD-MSCs have a therapeutic effect on lung injury caused by paraquat poisoning and reduce mortality and whether hAD-MSCs transplanted via veins can colonize and function in lung tissue. The research found that the peak concentration of plasma is attained within 4 h after intoxication in humans, and the pulmonary peak concentration is reached 4-5 h after i.v. administration in rats [12]. Meanwhile, according to the clinical experience of our hospital, patients who have ingested PQ involuntarily or who have used paraquat to attempt suicide tend to arrive at the hospital for treatment within 4 hours after ingesting the poison. Therefore, 4 hours, that is, the longest time spent to reach the hospital after the poisoning, is selected as the time of hAD-MSC transplantation. However, it is unclear whether stem cell transplantation has therapeutic effects on lung injury caused by paraquat poisoning at this time. Thus, further studies are necessary to address these issues.

## 2. Materials and Methods

### 2.1. Ethics Statement

Amniotic membranes delivered by caesarean section were obtained from women whose monotocous pregnancy had come to term. All women signed informed consent forms to donate the amniotic membrane. All women tested negative for hepatitis B virus (HBV), hepatitis C virus (HCV), HIV, and syphilis. This research was approved by the Ethics Committee of Zunyi Medical University. Animal studies were approved by the Animal Ethics Committee of Zunyi Medical University. Animals were cared for in accordance with the *Guide for the Care and Use of Laboratory Animals* in China.

### 2.2. Cell Isolation, Purification, Culture, and Identification

hAD-MSCs were isolated using protocols described as follows. Briefly, the human amniotic membrane peeled from fresh placenta was cut into pieces with scissors and, under sterile conditions, digested at 37°C for 30 min with 0.5 mg/ml trypsin (Gibco and Sigma-Aldrich, USA) and shaken continuously for 2 h with 0.75 mg/ml collagenase and 0.075 mg/ml DNAse I (Gibco and Sigma-Aldrich, USA) in a level shaker (200 rpm/min). After that, cells were washed with D-PBS containing penicillin and streptomycin (100 IU/ml and 0.1 mg/ml), resuspended in Dulbecco's modified Eagle medium with low glucose (1 g/L) and sodium pyruvate (LG-DMEM, Gibco, USA) with 10% fetal bovine serum (FBS, Gibco, USA), and placed in a 37°C incubator with saturated humidity and 5% carbon dioxide concentration in primary culture (passage 0 = P0). Cells at 80-90% confluency were harvested by treatment with 2.5 mg/ml trypsin and subcultured in the above medium. Third-passage (P3) cells were collected for phenotype analysis and cell transplantation. Third-passage hAD-MSCs, washed with D-PBS, were stained with the following antibodies: CD44, CD73, CD90, CD105, CD14, CD19, CD34, CD45 (cluster of differentiation, Becton Dickinson, USA), and HLA-DR (human leukocyte antigen DR, Becton Dickinson, USA) and corresponding isotype controls. Staining was performed according to the manufacturer's instructions. Analysis was performed using the FACSCalibur cytometer (Becton Dickinson, USA).

### 2.3. Animal Model and Cell Transplantation

Forty Sprague-Dawley male rats (200 ± 20 g) were purchased from the Experimental Animal Center of Daping Hospital, Army Medical University (Third Military Medical University) (license no. SCXK2012-0005) and were maintained under pathogen-free conditions. Rats were fasted for 12 h before the experiment. Three groups, defined as the sham control group (*n* = 8), model group (*n* = 15), and hAD-MSC-transplanted group (*n* = 17), were formed by randomly dividing rats among the groups. Rats in the model group and the hAD-MSC-transplanted group received an intraperitoneal injection [24] of 20% paraquat (Kexin, Shandong, China) aqueous solution (30 mg/kg). The sham control group received an intraperitoneal injection of normal saline. Rats in the hAD-MSC-transplanted group were administered 200 *μ*l of hAD-MSC suspension in LG-DMEM medium (~1 × 10^6^ cells, approximately 5 × 10^6^ cells per kilogram body weight) by injection via the sublingual vein. Intravenous injection is more suitable for clinical patients. Rats in the model and sham control groups were injected with 200 *μ*l LG-DMEM medium. Rats were observed seven days after PQ exposure to evaluate the therapeutic effectiveness of cell transplantation. At the completion of experiment, all surviving rats were euthanized by asphyxiation with CO_2_, following the procedures recommended by the Panel on Euthanasia of the American Veterinary Medical Association.

### 2.4. Survival Analysis

The activity, food weights, and breathing frequency of rats were observed daily for seven days after treatment with PQ, and Kaplan-Meier survival curves were estimated.

### 2.5. Histological Staining

#### 2.5.1. H&E Staining

The lower right lung of each rat was collected (survival in the model group: *n* = 6; survival in hAD-MSC-transplanted group: *n* = 13) on the 7^th^ day after hAD-MSC transplantation. The lower right lung samples were then immersion-fixed in 4% paraformaldehyde for 24 h. After fixation, the samples were embedded in paraffin and cut into sections of 5 *μ*m thickness. First, paraffin sections were dried in an oven at 60°C and deparaffinized in xylene and rehydrated using gradient alcohol (100-95-85-75%). Samples were stained with hematoxylin for 2 min, rinsed in 1% hydrochloric acid for 10 sec, and then placed in 75% alcohol for 10 sec, and rinsed with tap water for 10 sec; stained with eosin for 5 min; and placed in sequential alcohol solution in a gradient (85-95-100%) and xylene for dehydration; samples were then coverslipped with neutral balsam.

#### 2.5.2. Masson's Trichrome Staining

After the above paraffin sections were deparaffinized in xylene and rehydrated using gradient alcohol (100-95-85-75%), the samples were rinsed with 30-40°C hot water twice, each time 60 sec. Following the protocol and instructions of the Masson staining kit (HyClone, Logan, USA), sections were stained with Masson's trichrome and coverslipped with neutral balsam.

#### 2.5.3. Sirius Red Staining

After the paraffin sections were deparaffinized in xylene and rehydrated using gradient alcohol (100-95-85-75%), samples were stained with the Sirius red staining kit (HyClone, Logan, USA) and coverslipped with neutral balsam.

#### 2.5.4. Histologic Score

Histologic scores of the above histological staining were obtained independently by two experienced pathologists.

#### 2.5.5. ELISA Detection of TGF-*β*1, HYP, and HLA-G5 in Serum

Serum was collected (sham control group: *n* = 8; model group: *n* = 6; and transplanted group: *n* = 13) on day 7 after transplantation. The concentrations of TGF-*β*1, HYP, and HLA-G5 were measured using respective ELISA kits (TSZ, Framingham, USA).

#### 2.5.6. Immunohistochemistry and Hematoxylin Staining to Detect Human Cells

After the above paraffin sections were deparaffinized in xylene and rehydrated using gradient alcohol (100-95-85-75%), the samples were incubated in 0.3% TritonX-100 for 20 min, followed by 3% H_2_O_2_, and were incubated at room temperature for 10 min in darkness. Sections were then rinsed in PBS for 5 min three times, blocked in 10% goat serum for 30 min, and then incubated with the HLA-G5 antibody (Epitomics, California, USA) or isotype control antibody at 4°C overnight, followed by 35-minute PBS washes. Sections were then incubated with the second antibody of the EnVision™ Detection Kit (DAKO, California, USA) and incubated at room temperature for 1 h, rinsed with PBS for 5 min three times, stained with DAB for 3-5 min, washed with tap water for 30 sec, stained with hematoxylin for 30 sec, rinsed with tap water, put through an alcohol gradient dehydration (100-95-85-75%), vitrificated by dimethylbenzene, enclosed in neutral balsam, and photographed under a microscope.

#### 2.5.7. Statistical Methods

SPSS 17.0 software was used for statistical analysis. Experimental data was presented as mean ± SEM, and the groups were compared using the independent sample *t*-test and Kaplan-Meier survival analysis using the log-rank (Mantel-Cox) test. A *p* < 0.05 was considered statistically significant.

## 3. Results

### 3.1. Characterization of hAD-MSCs

**Figure 1:**
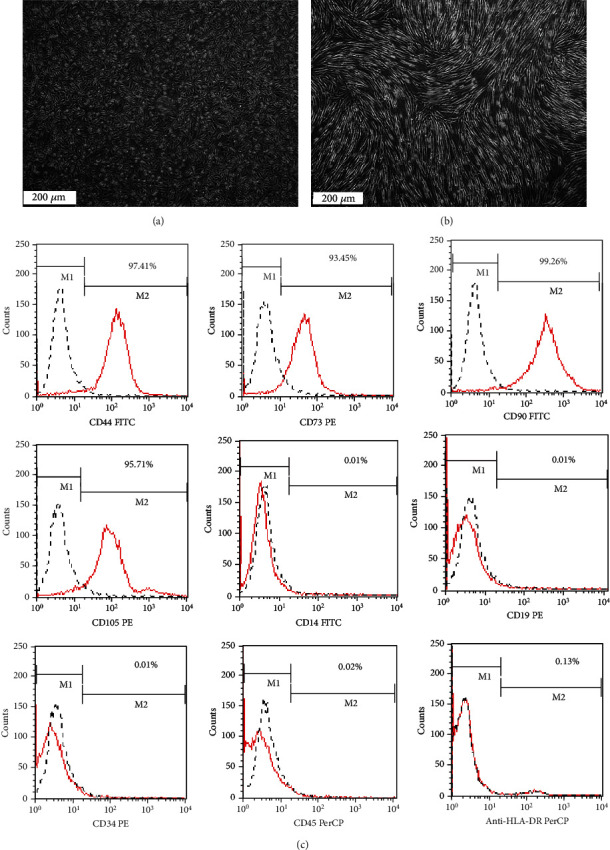
Morphology of cultured hAD-MSCs and phenotypic analysis of purified hAD-MSCs by FCM. (a) Primary hAD-MSCs with diverse morphology. (b) Third-passage purified hAD-MSCs with fibroblast-like morphology. The scale is 200 *μ*m. (c) Purified hAD-MSCs exhibited classic phenotypic hallmarks of MSCs, stained strongly positive for CD44, CD73, CD90, and CD105 and stained negative for CD14, CD19, CD34, CD45, and HLA-DR.

Human amnion-derived mesenchymal stem cells displayed adherent growth characteristics of primary culture cells. The cell morphology was diverse polygon, spindles, and other shapes. After purification, the morphology of the 3^rd^ passage of hAD-MSCs was similar to that of fibroblasts. The hAD-MSCs exhibited classic phenotypic hallmarks of MSCs (found via flow cytometric (FCM) analysis), stained strongly positive for CD44, CD73, CD90, and CD105 and negative for CD14, CD19, CD34, CD45, and HLA-DR, one of the HLA-II molecules (Figure 1).

### 3.2. Human Amnion-Derived Mesenchymal Stem Cell Transplantation Increased the Survival Rate

**Figure 2:**
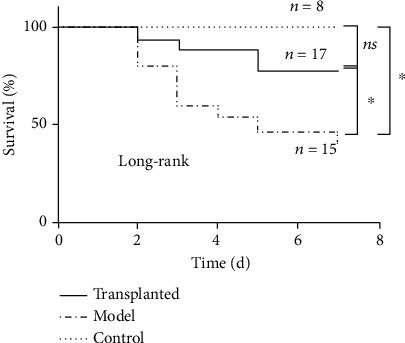
Survival analysis. All rats in the model group (*n* = 15) and transplanted group (*n* = 17) after PQ poisoning were observed daily for 7 days. The sham control group (*n* = 8) was observed at the same time, and the survival curves were drawn. Kaplan-Meier survival analysis using the log-rank (Mantel-Cox) test (^∗^*p* < 0.05).

To test the main efficacy of hAD-MSC transplantation, the state of being and survival of rats after PQ poisoning were observed for seven days. Compared with the sham control group and the hAD-MSC-transplanted group, rats in the model group showed a distinct reduction in activity and food weight. Furthermore, rats in the model group had an increased breathing frequency 24-48 h post-PQ injection. As expected, a significant difference in the survival rate of rats between the model and sham control group was observed (*p* < 0.05). The survival rate of hAD-MSC-transplanted group rats, 76.5% in seven days, showed no significant difference compared with the sham control group (100% survival, *p* = 0.25). Survival of rats given hAD-MSC transplantation was significantly more likely than rats in the model group (survival rate 40%, *p* < 0.05). This data is shown in Table 1 and Figure 2. The results suggest that hAD-MSC transplantation significantly improves the survival of rats poisoned with PQ.

### 3.3. Human Amnion-Derived Mesenchymal Stem Cell Transplantation Ameliorated PQ-Induced Pulmonary Injury and Inflammation

**Figure 3:**
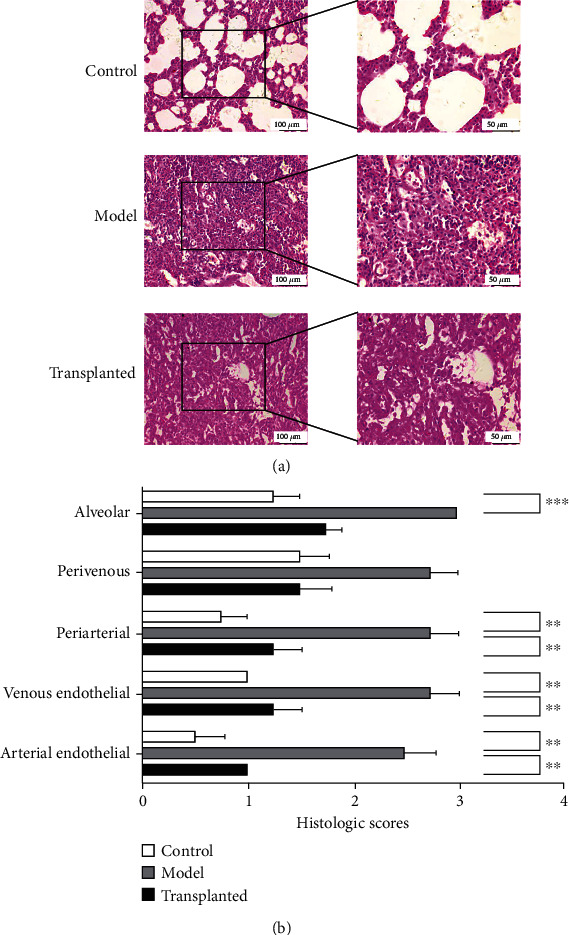
H&E staining of lung tissue. (a) Rats in the model group (*n* = 6) showed significant pulmonary interstitial thickening, mutual integration between alveoli, significant damage to the structure, disappearance of the original alveolar structure, a large number of interstitial lung telangiectasia, associated diffuse pulmonary hemorrhage, pulmonary capillary, and alveolar spaces surrounding visible inflammatory cell infiltration. Rats in the hAD-MSC-transplanted group (*n* = 13) also showed alveolar structural damage, fusion, a small amount of interstitial pulmonary capillary congestion, exudation around a small number of red blood cells, and pulmonary capillaries and alveoli around inflammatory cells. Inflammatory cell infiltration was significantly less than that of the model group. The scale is 100 *μ*m under low magnification view and 50 *μ*m under high magnification view. (b) Histologic scores were obtained blindly using previously published criteria [25]. Each bar represents the mean ± SEM (^∗∗^*p* < 0.01, ^∗∗∗^*p* < 0.001). Not marked if not significantly different.

To examine the severity of lung injury, the inflammatory infiltration, and the extent of fibrosis, histological staining was implemented. H&E staining of lung tissue harvested from rats in the model group showed significant pulmonary interstitial thickening, mutual integration between the alveoli, significant damage to the structure, disappearance of the original alveolar structure, a large number of interstitial lung telangiectasia, associated diffuse pulmonary hemorrhage, and pulmonary capillary and alveolar spaces surrounding visible inflammatory cell infiltration compared to sham control rats. The hAD-MSC-transplanted rats also appeared to have alveolar structural damage, fusion, a small amount of interstitial pulmonary capillary congestion, and exudation around a small number of red blood cells compared to the model rats. However, inflammatory cell infiltration in rats given hAD-MSC transplantation was significantly less than that in the model group. The histologic scores of the transplanted group also significantly exceeded those of the model group. These results indicate that hAD-MSCs can not only protect alveolar epithelial cells and alleviate lung injury but also inhibit inflammation (Figure 3).

### 3.4. Human Amnion-Derived Mesenchymal Stem Cell Transplantation Hindered PQ-Induced Pulmonary Fibrosis Progression

**Figure 4:**
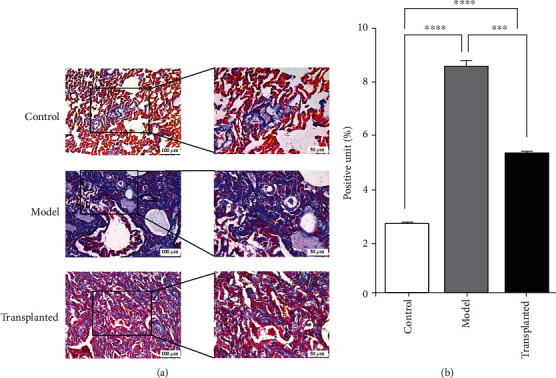
hAD-MSCs hinder PQ-induced pulmonary collagen deposition. Masson's trichrome staining of lung tissue. Collagen fibers were dyed blue by Masson's trichrome stain. (a) The collagen deposition is significantly increased in the model group rats (*n* = 6) compared to the model group rats (*n* = 8) but reduced in the hAD-MSC-transplanted group rats (*n* = 13). The scale is 100 *μ*m under low magnification view and 50 *μ*m under high magnification view. (b) Collagen fibers were dyed blue and quantified by using ImageJ. ^∗∗∗^*p* < 0.001, ^∗∗∗∗^*p* < 0.0001.

Masson's trichrome staining of samples taken from rats in the model group showed more extensive damage to the alveolar space, a significant amount of alveolar interstitial fibroblast proliferation, and alveolar wall thickening. Samples taken from rats in the hAD-MSC-transplanted group also appeared to have slight alveolar structural damage and a small amount of interstitial pulmonary capillary congestion (Figures 4(a) and 4(b)).

**Figure 5:**
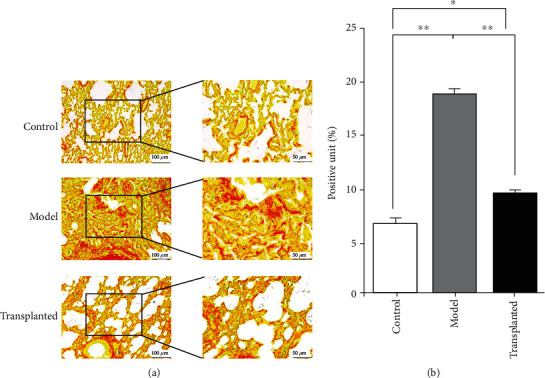
hAD-MSCs reduce PQ-induced pulmonary type I collagen deposition. Sirius red staining of lung tissue. (a) Red staining on light microscopy is type I collagen deposition, which is significantly increased in the model group rats (*n* = 6) compared to the model group rats (*n* = 8) but reduced in the hAD-MSC-transplanted group rats (*n* = 13). The scale is 100 *μ*m under low magnification view and 50 *μ*m under high magnification view. (b) Type I collagen fibers were dyed red and quantified by using ImageJ. ^∗^*p* < 0.05, ^∗∗^*p* < 0.01.

Similar results were observed with Sirius red staining: a large number of alveoli with significant damage to the original structure, a large amount of fibroblast proliferation, and diffuse pulmonary interstitial thickening were observed in samples from rats in the model group, while samples taken from hAD-MSC-transplanted rats presented fewer phenotypic characteristics of fibrosis progression (Figures 5(a) and 5(b)).

**Figure 6:**
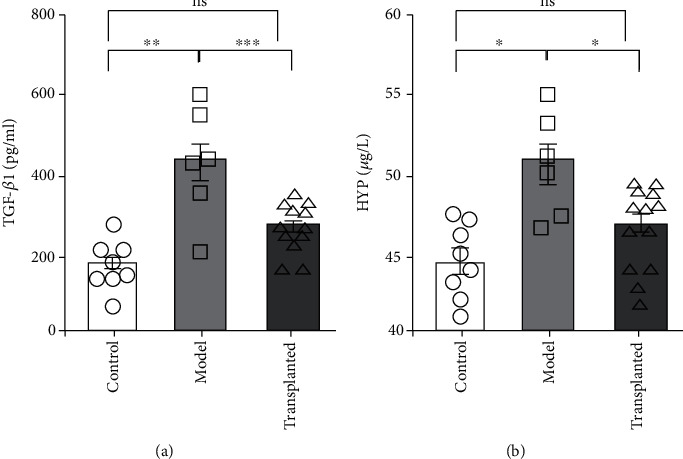
hAD-MSCs reduce the cytokine that promotes collagen formation and collagen fiber. TGF-*β*1 (a) and HYP (b) in serum were measured by ELISA. TGF-*β*1, a major profibrotic cytokine, and HYP, the main component of collagen fibers, were measured using an ELISA kit. TGF-*β*1 levels increased following PQ exposure, accompanied by the increased HYP serum concentration in the model group (*n* = 6) compared to the sham control group (*n* = 8). TGF-*β*1 and HYP levels decreased following hAD-MSC transplantation (*n* = 13). Statistical analysis was performed using the unpaired *t*-test. ^∗^*p* < 0.05, ^∗∗^*p* < 0.01, and ^∗∗∗^*p* < 0.001. ns = not significant.

TGF-*β*1 is a major profibrotic cytokine. Hydroxyproline (HYP) is the main component of collagen fibers, and therefore, serum HYP can be used as an indicator of lung fibrosis. TGF-*β*1 and HYP in serum were measured by ELISA as indicators of fibrosis. TGF-*β*1 levels were enhanced following PQ exposure in rats of the model group compared to the sham control group (Figure 6(a)). HYP in the serum of model group rats was also increased compared to that of the sham control group (Figure 6(b)). Interestingly, TGF-*β*1 and HYP levels decreased following treatment with hAD-MSCs compared to control. This indicates that hAD-MSCs hindered the process of pulmonary fibrosis.

### 3.5. Survival of hAD-MSCs and Secretion of the Immunosuppressive Factor HLA-G5

**Figure 7:**
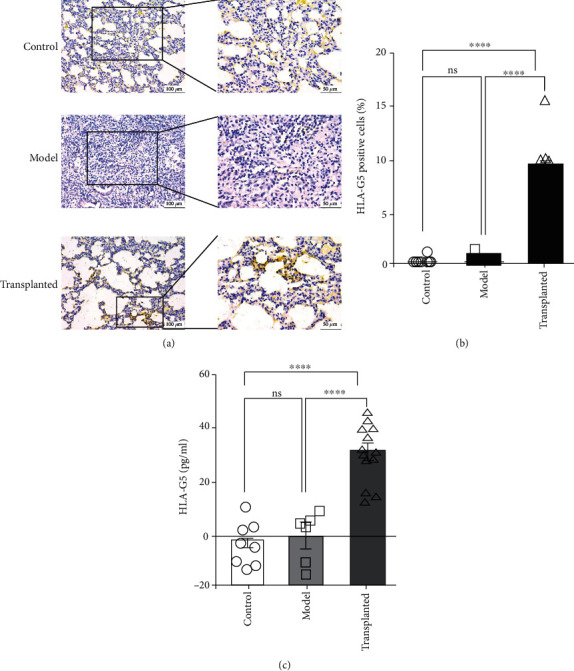
Survival of hAD-MSCs in the damaged lung and concentration of HLA-G5 in serum. (a) Human leukocyte antigen G5 was stained with antibody HLA-G5. Hematoxylin was used to redye cell nuclei. HLA-G5-positive cells were not observed in the lung from the control group (*n* = 8) and model group (*n* = 6), but the hAD-MSC-transplanted group (*n* = 13). The scale is 100 *μ*m under low magnification view and 50 *μ*m under high magnification view. (b) The HLA-G5-positive cells were quantified per 100 cells. (c) Secretion of the immune suppressor HLA-G5 in serum was measured using an ELISA kit. HLA-G5 in rats from the hAD-MSC-transplanted group (*n* = 13) was remarkably higher than that in the control group (*n* = 8) and model group (*n* = 6). There was almost no difference in HLA-G5 levels between the other two groups in serum. Statistical analysis was performed using the unpaired *t*-test. ^∗∗∗∗^*p* < 0.0001. ns = not significant.

The soluble HLA-G5 is the predominant isoform secreted by MSCs and may act as a crucial immunosuppressive molecule for T cells, natural killer cells, dendritic cells, and other immune cells. To explore the mechanisms of anti-inflammation and immunosuppression in all groups, we examined levels of the immune suppressor HLA-G5 and the survival of hAD-MSCs. We determined that hAD-MSCs survived in the PQ-damaged lung via detection of the HLA-G5, which is species specific (Figures 7(a) and 7(b)). Moreover, we determined that the secretion of the immune suppressor HLA-G5 in serum was remarkably higher in rats with hAD-MSC transplantation than in rats in the sham control and model groups (Figure 7(c)). There was almost no detection between the latter groups in serum. These findings suggest that HLA-G5 is likely a crucial factor involved in enabling hAD-MSC-mediated anti-inflammation and immunosuppression. This shows that the hAD-MSCs are not only surviving but also performing a secretory function to suppress immune rejection.

## 4. Discussion

There is currently no effective clinical therapy for PQ poisoning [8, 11]. Research has shown that ALI is the leading cause of acute and chronic death as a result of pulmonary fibrosis [12, 13]. The main aim of our research was, therefore, to treat ALI and stop fibrosis. Current treatments generally involve cytoprotection, in which dialysis or gastric lavage is first used to reduce pesticide residues, followed by medication, that is, anti-inflammatory or immunosuppressive and antioxidants [14, 16]. An increasing number of studies have shown that MSCs can repair damaged lung tissue and inhibit inflammation [15]. The results of the present study demonstrated that hAD-MSCs exhibited the classic phenotypic hallmarks of MSCs, stained positive for CD44, CD73, CD90, and CD105 and negative for hematopoietic stem cell markers including CD14, CD19, CD34, and CD45, as well as negative for HLA-DR, which is one of the HLA class II molecules. Given the unique characteristics of hAD-MSCs, we hypothesized that hAD-MSC transplantation could be used as an effective therapy for PQ poisoning.

In this study, we attempted to recapitulate clinical conditions and evaluated the preclinical efficacy of hAD-MSC transplantation to treat PQ-induced ALI and pulmonary fibrosis. We first characterized hAD-MSCs. Subsequently, the state of being, survival, pneumonocyte damage, infiltration of inflammatory cells, and extent of fibrosis were evaluated using direct microscopy of histological staining. Rats surviving seven days post-PQ poisoning will usually survive a longer term and only show begining with pulmonary fibrosis [5, 13]. Thus, the seventh day posttransplantation was chosen as the end of this experiment. In an attempt to illustrate the mechanism of this phenomenon, we investigated the crucial profibrotic factor TGF-*β*1, a major component of fibrotic collagen, HYP, and HLA-G5, the crucial immunosuppressive factor secreted by MSCs [26]. The hAD-MSCs used for transplantation presented typical hallmarks of MSC adherence and fibrous growth. The hAD-MSC transplantation significantly improved the state of being and survival of PQ-poisoned rats. In addition, lung injury, inflammation, and profibrotic factors were noticeably reduced. Our results also showed a remarkable increase in serum HLA-G5, a critical immunosuppressive molecule for T cells, natural killer cells, dendritic cells, and other immune cells [26, 27], which indicated that hAD-MSCs might be effective for the treatment of PQ poisoning via anti-inflammation, immunosuppression, and downregulation of profibrotic factors. Therefore, our study revealed that hAD-MSC transplantation may be a new therapeutic approach for the treatment of PQ-intoxicated patients, largely mediated through secretory immune factors and immunoregulation. Further investigations on the cytoprotection and repair of damaged lung cells and oxidative resistance are underway. However, whether they modulate or activate lung progenitors by survival hAD-MSCs located at the bronchoalveolar duct junction remains to be elucidated.

Human bone marrow-derived MSCs administered intravenously can engraft in animals because MSCs do not provoke an immune reaction and therefore evade host immune elimination [28, 29]. At the same time, hAD-MSCs were a newly discovered mesenchymal stem cell. Based on these facts, we used hAD-MSCs to treat immune-competent SD rats. Similarly, we identified the phenotypic characteristics and low immunogenicity of hAD-MSCs by FCM and determined that the survival rate of PQ-poisoned rats increased. The symptoms of lung injury were reduced posttransplantation. Previous studies also found that MSCs exhibit cytoprotective effects through antioxidative, anti-inflammatory, and antiapoptotic actions. Although our study showed no antioxidant and inflammatory factors, i.e., MDA, SOD, GSH-Px, TNF-*α*, IL-6, and IL-1*β*, the effects of anti-inflammation and antifibrosis were identified [5, 19, 20, 30]. We found the reduction of inflammatory cell infiltration and pulmonary edema, which account for the appearance of anti-inflammation. Moreover, the reduced anti-inflammatory factor IL-10 and increased inflammatory factor IL-17 posttransplantation were also detected (Supplementary Figure 1) for further elucidation of key anti-inflammatory and inflammatory factors. T regulatory type 1 (TR1) cells secrete high levels of the anti-inflammatory IL-10 [31], whereas TH17 cells are characterized by secretion of IL-17 [32]. TGF-*β*1 supports the conversion of TH17 to TR1 [33, 34]. Taken together, these results indicate that TH17 cells might transdifferentiate into TR1 cells induced by a high concentration of TGF-*β*1 in the model group, and the decrease in the concentration of TGF-*β*1 leads to a decrease in the conversion of TH17 to TR1, resulting in an increase in the concentration of TH17 and a decrease in its concentration. We detected HLA-G5 expression *in vivo*, and HLA-G5 was highly expressed consistently with the reduction of TGF-*β*1 after hAD-MSC transplantation. Thus, it needs to be further verified whether HLA-G5 prevents TH17 cell transdifferentiation through inhibiting the secretion of TGF-*β*1, which can promote fibrosis and immunoregulation. Further mechanisms may be considered about hAD-MSCs inhibiting inflammation and preventing fibrosis. HLA-G5 has inhibitory effects on several immune cells [35, 36]; further determination of cell types and the cytokine profile is warranted in follow-up studies with FCM analysis and detection of inflammatory factors [37, 38].

Unlike other stem cells such as previous bone marrow MSCs, human amnion-derived MSCs are a postnatal discard, noninvasive, and less ethically controversial and offer significant advantages. Furthermore, there is no effective treatment for lung injury specifically targeted by paraquat, for which human amnion-derived MSCs are remarkably effective.

In this study, we provide an effective stem cell therapy for paraquat poisoning and find that it plays an important role in reducing acute mortality, mitigating acute lung injury and delaying pulmonary fibrosis after paraquat poisoning in rats, providing a new therapeutic strategy for people who have accidentally ingested or have been occupationally exposed to paraquat.

## Figures and Tables

**Table 1:** Effect of hAD-MSC transplantation on survival of rats with PQ poisoning.

Groups	Death (*n*)	Survival (*n*)	Survival rate (%)
Control	0	8	100
Model	9	6	40.0^∗^
Transplanted	4	13	76.5^∗^^▲^

^∗^Compared to the sham control group (*p* <0.05); ^▲^compared to the model group (*p* <0.05); between the sham control group and the transplanted group: *p* = ns, ns = not significant.

## Data Availability

The authors confirm that the data supporting the findings of this study are available within the article and its supplementary materials.

## Supplementary Materials

Supplementary Materials Supplementary Table 1: effect of hAD-MSC transplantation on survival of rats with PQ poisoning. Supplementary Table 2: the time of death for each group of rats. Supplementary Figure 1: concentration of IL-10 (A) and IL-17 (B) in serum.
