# Supplementary material for: Human Amnion-Derived MSCs Alleviate Acute Lung Injury and Hinder Pulmonary Fibrosis Caused by Paraquat in Rats
Source: Oxid Med Cell Longev. 2022 Mar 19;2022:3932070. doi: 10.1155/2022/3932070 (PMC8957415; doi:10.1155/2022/3932070)
Supplement: Supplementary Materials — Supplementary Table 1: effect of hAD-MSC transplantation on survival of rats with PQ poisoning. Supplementary Table 2: the time of death for each group of rats. Supplementary Figure 1: concentration of IL-10 (A) and IL-17 (B) in serum. [file 3932070.f1.pdf]

Highlights:

hAD-MSCs improve the survival rate of paraquat-induced acute poisoning rats.

hAD-MSCs can reduce acute lung injury and delay chronic pulmonary fibrosis both paraquat-induced.

hAD-MSCs can survive in situ in the lung and secrete HLA-G5 to the serum, which may affect the progression of inflammation and fibrosis through immunoregulation.

Supplementary Tab.1

Effect of hAD-MSCs transplantation on survival of rats with PQ poisoning

Groups	death(n)	survival(n)	survival rate(%)
Control	0	8	100
Model	9	6	40.0*
Transplanted	4	13	76.5*▲

*Compared with the normal control group, $p < 0.05$; ▲compared with the model group, $p < 0.05$; between the normal control group and the transplanted group, $p=0.2451$.

Supplementary Tab.2

The time of death for each group of rats

Groups	Day1	Day2	Day3	Day4	Day5	Day6	Day7	(death/total)
Control	0	0	0	0	0	0	0	(0/8)
Model	0	3	3	1	1	0	1	(9/15)
Transplanted	0	1	1	0	2	0	0	(4/17)

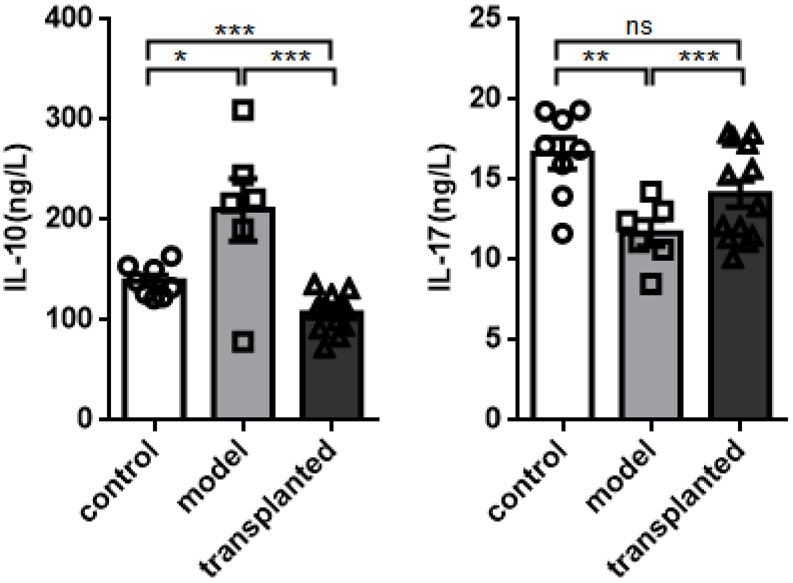

A B

Supplementary Figure.1 Concentration of IL-10 (A) and IL-17 (B) in serum. Secretion of immune inflammatory factor IL-10 and IL-17 in serum was measured using an ELISA kit. IL-10 in rats from the model group (n=6) was remarkably higher than in the normal control group (n=8) and hAD-MSCs-transplanted group (n=13), and the level in the hAD-MSCs-transplanted group lower than in the normal control group. To the contrary, IL-17 level was the lowest in the model group in serum, and the level in the hAD-MSCs-transplanted group lower than in the normal control group. Statistical analysis was performed using the unpaired t test. $*p<0.05, **p<0.01, ***p<0.001$.
